# Current classification of zirconia in dentistry: an updated review

**DOI:** 10.7717/peerj.15669

**Published:** 2023-07-14

**Authors:** Suchada Kongkiatkamon, Dinesh Rokaya, Santiphab Kengtanyakich, Chaimongkon Peampring

**Affiliations:** 1 Department of Prosthetic Dentistry, Faculty of Dentistry, Prince of Songkla University, Songkhla, Thailand; 2 Faculty of Dentistry, Zarqa University, Zarqa, Jordan; 3 Prosthodontic Section, Department of Restorative Dentistry, Naresuan University, Phitsanulok, Thailand

**Keywords:** Dental ceramic, Y-TZP, 3Y-TZP, 5Y-TZP, CAD/CAM, Translucency

## Abstract

Zirconia, a crystalline oxide of zirconium, holds good mechanical, optical, and biological properties. The metal-free restorations, mostly consisting of all-ceramic/zirconia restorations, are becoming popular restorative materials in restorative and prosthetic dentistry choices for aesthetic and biological reasons. Dental zirconia has increased over the past years producing wide varieties of zirconia for prosthetic restorations in dentistry. At present, literature is lacking on the recent zirconia biomaterials in dentistry. Currently, no article has the latest information on the various zirconia biomaterials in dentistry. Hence, the aim of this article is to present an overview of recent dental zirconia biomaterials and tends to classify the recent zirconia biomaterials in dentistry. This article is useful for dentists, dental technicians, prosthodontists, academicians, and researchers in the field of dental zirconia.

## Introduction

Zirconia (ZrO_2_) is a crystalline oxide of zirconium and it holds good mechanical, optical, and biological properties (Bapat et al., 2022). This biomaterial has three basic chemical forms; monoclinic, tetragonal, and cubic (Saridag, Tak & Alniacik, 2013; Bocanegra-Bernal & dela Torre, 2002). The metal-free restorations, mostly consisting of all-ceramic/zirconia restorations, are becoming popular restorative materials in restorative dentistry choices for aesthetic and biological reasons (Kongkiatkamon et al., 2021). Recently, there have been significant improvements in restorative biomaterials including dental zirconia, and producing wide varieties of zirconia for prosthetic restorations in dentistry (Kontonasaki et al., 2019; Kontonasaki, Giasimakopoulos & Rigos, 2020; Humagain & Rokaya, 2019; Amornvit et al., 2021). With the advancement of digital technologies, intraoral scanners, and CAD/CAM systems, it has become possible to fabricate dental restorations digitally with easy processing, designing, and high accuracy (Al-Qahtani et al., 2021; Ahmed et al., 2021).

**Figure 1:**
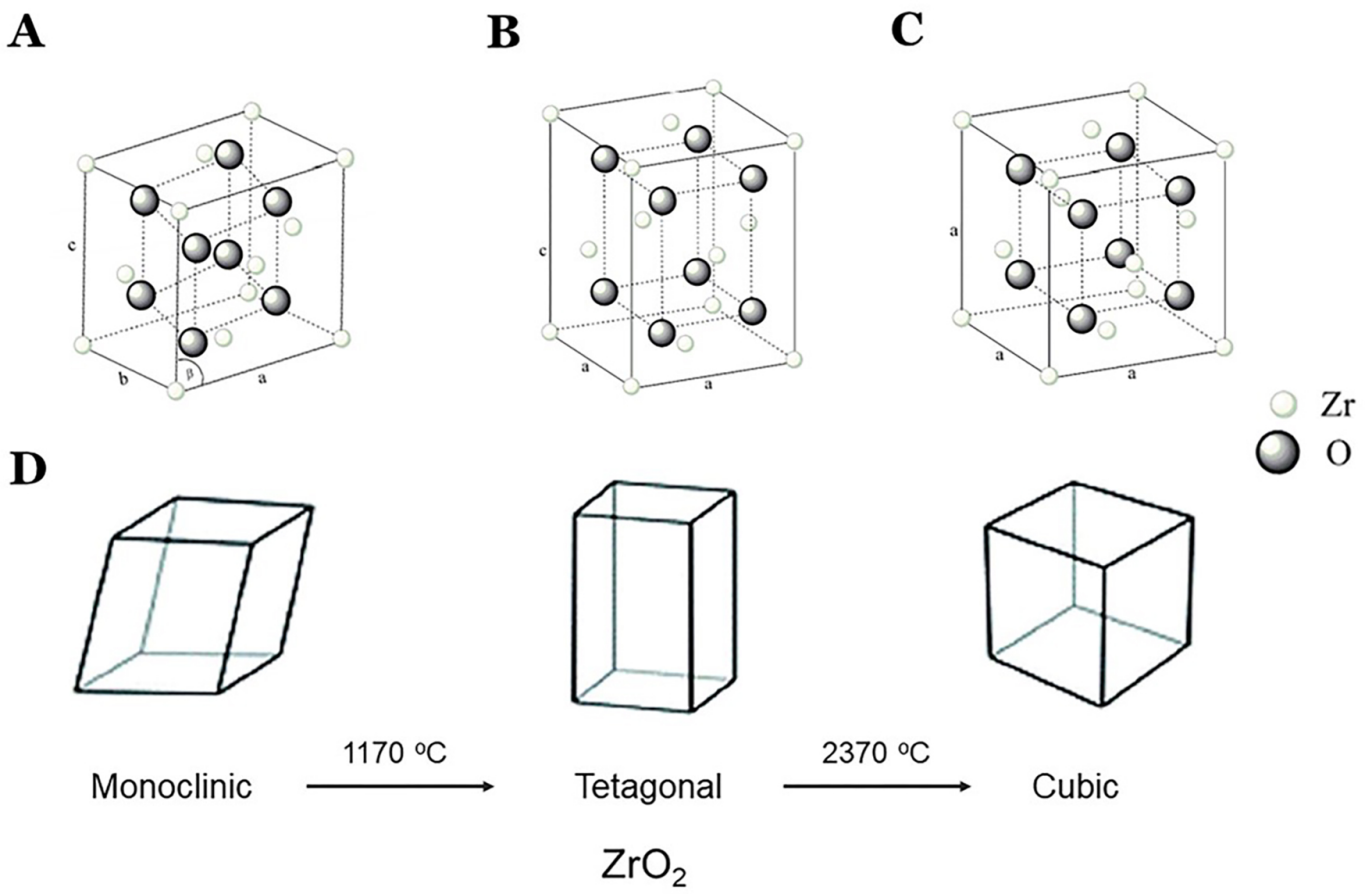
Phases and its transformation of zirconia. (A) monoclinic; (B) tetragonal; (C) cubic structure; and (D) phases of transformation of zirconia. Modified with permission from Sorrentino et al. (2019).

Pure zirconia exists in the monoclinic form at room temperature and with an increase in temperature (1,170 °C) or low-temperature degradation (LTD), it transforms to the tetragonal form (Ban, 2021). Further increasing temperature (2,370 °C), aging or hydrothermal aging, progressive transformation to monoclinic phase takes place (Piconi & Maccauro, 1999; Sorrentino et al., 2019; Rekow et al., 2011) (Fig. 1). Then cooling, the tetragonal form transforms back to the monoclinic form. Achieving stable sintered zirconia ceramic is a little difficult because volumetric change (about 5%) occurs when the transformation from tetragonal to monoclinic. The zirconia can be monochromatic with uniform composition, polychromatic multilayer with uniform composition, and polychromatic multilayer and hybrid composition.

Proper bonding between the zirconia restoration and the tooth is important for the longevity of the prosthetic restoration (Araújo et al., 2018; Melo et al., 2015; Heboyan et al., 2023). Zirconia requires surface treatments with acid etching for surface abrasion to ensure adhesion with luting cement (Araújo et al., 2018). Although there are various surface treatment protocols have been recommended, common treatment included alumina particles followed by the application of primers or cements based on MDP (10-methacryloyloxydecyl dihydrogen phosphate) (Melo et al., 2015; Aung et al., 2019; Shimizu et al., 2018; Silveira et al., 2022; Alammar & Blatz, 2022). The surface modification improves the adhesive behavior of the materials (Silveira et al., 2022).

Dental zirconia has increased over the past years producing wide varieties of zirconia for prosthetic restorations in dentistry. Although some researchers have studied zirconia and classified dental zirconia in the past, (Ban, 2021; Güth et al., 2019; Nistor et al., 2019; Grech & Antunes, 2019; Alqutaibi et al., 2022) the current literature is lacking on the recent zirconia biomaterials in dentistry. The research question is there a recent classification of the recent zirconia biomaterials in dentistry? It is found that no article has the latest information on the various types of zirconia biomaterials in dentistry. Hence, the aim of this article is to present an overview of recent dental zirconia biomaterials and tends to classify the recent zirconia biomaterials in dentistry. This article is useful for dentists, dental technicians, prosthodontists, and researchers in the field of dental zirconia by providing updated information on the current literatures on various types of zirconia used in dentistry.

## Survey Methodology

Articles on advances in dental zirconia ceramic were searched from January 1989 to December 2022 using Google Scholar, MEDLINE/PubMed, Web of Science, and ScienceDirect resources. Research and review articles in the English language were only included in this review. A total of 79 articles were selected and included in this review. Editorials, Letters to the Editor, and Case Reports were excluded from this review.

### Yttria stabilized zirconia

Often in zirconia, various elements are dissolved such as yttrium (Y), cerium (Ce), calcium (Ca), magnesium (Mg), etc. to make it stable at room temperature (Piconi & Maccauro, 1999; Chevalier, 2006). The addition of Yttria (Y_2_O_3_) to zirconia stabilizes the tetragonal phase (Leib et al., 2015). Following LTD, yttria is exhausted through reaction causing the phase transformation (Rekow et al., 2011; Chevalier, Cales & Drouin, 1999; Amat et al., 2019). Yttria-doping can reduce grain growth, stabilize the tetragonal phase, and substantially improve thermal stability. Furthermore, the thermal stability of the cubic form of zirconia is obtained by the substitution of some Zr4+ ions (ionic radius of 0.82 Å) with larger ions, *e.g.*, Y3+ (ionic radius of 0.96 Å) in the crystal lattice. This doping of zirconia results in partially stabilized zirconia (PSZ) (Leib et al., 2015).

**Figure 2:**
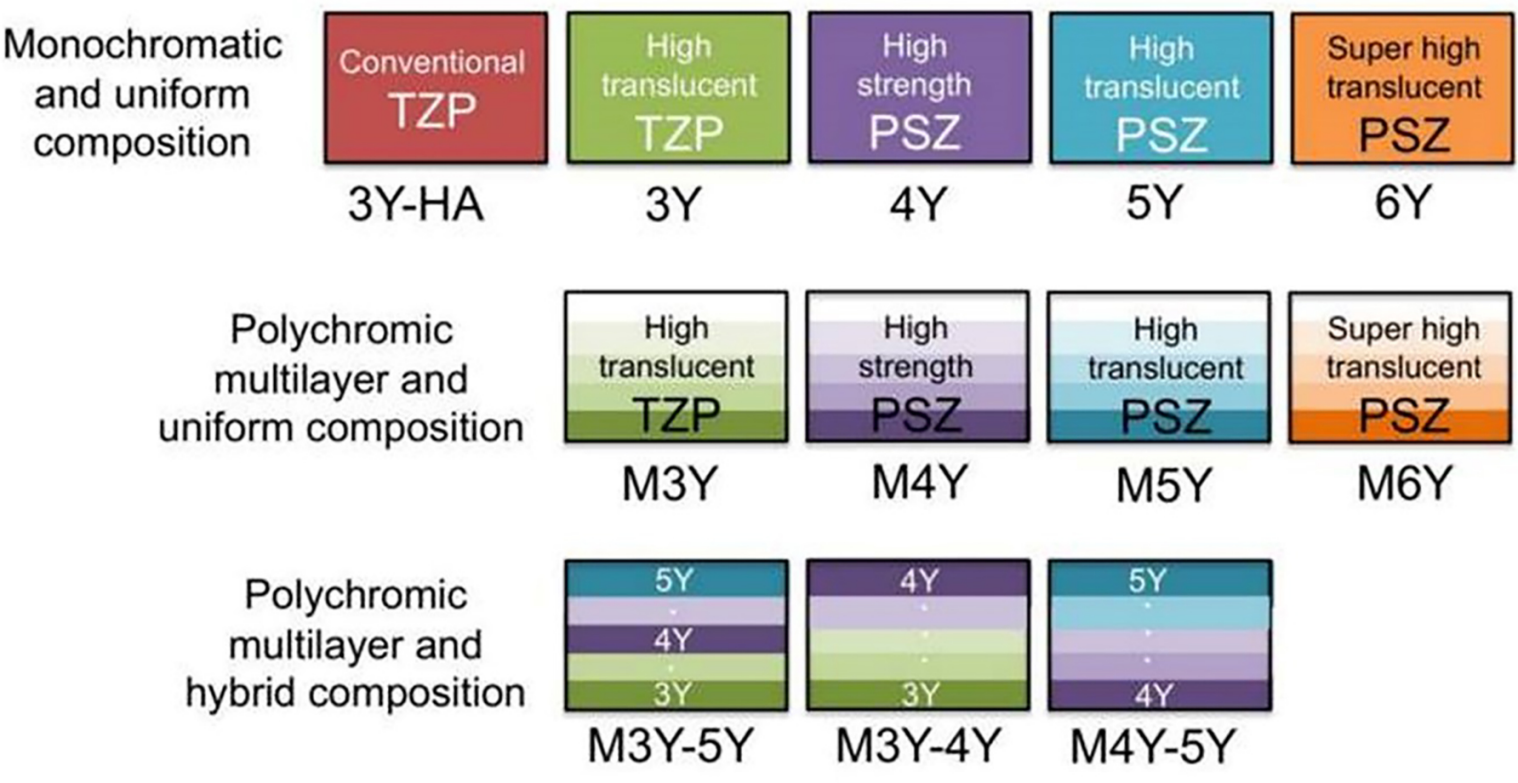
Classification of yttria-stabilized dental zirconia Ban (2021). Y, Yttria, M, multilayer.

The yttria-stabilized dental zirconia is classified into 12 types (Fig. 2). Zirconia (TZP, tetragonal *zirconia* polycrystal) are of various types based on the yttria content: (Zhang, 2014; Abdulmajeed et al., 2020; Arcila et al., 2021) 3Y-TZP (3 mole % Y-TZP), 4Y-TZP (4 mole % Y-TZP), 5Y-TZP (5 mole % Y-TZP), and 6Y-TZP (6 mole % Y-TZP). The 3Y-TZP is early zirconia used in dentistry as a “white metal” (Miyazaki et al., 2013). Zirconia with lower yttria content (3Y-TZP, 3 mole % Y-TZP) has better mechanical properties and less translucency whereas 3Y-TZP (3 mole % Y-TZP) with increased yttria content (6Y-TZP, 6 mole % Y-TZP) has more translucency but presents lower mechanical properties. Yttria content consisting of >8 mol% has a stable cubic phase at room temperature and it is known as cubic stabilized zirconia (CSZ). Similarly, yttria content consisting of 3-8 mol% has tetragonal and cubic phases and it is known as partially stabilized zirconia (PSZ). And yttria content consisting of approx. 3 mol% has tetragonal phases (toughened) about 100% and it is known as a tetragonal zirconia polycrystal (TZP).

At present multilayer (M) zirconia has been introduced. Similarly, M3Y is highly translucent and M6Y is super highly translucent (Fig. 2). Some surface defects can be seen in all types of zirconia under scanning electron microscopy, although the 3Y-TZP demonstrates higher grain consistency. It has been found that the 5Y-PSZ presents the least strength and the 4Y-PSZ and 3Y-TZP present similar fatigue. It has been found that higher yttria content has lower mechanical strength but higher translucency of zirconia (Ban, 2021; Harada et al., 2020; Cho et al., 2020). Similar to yttria, ceria (CeO_2_) is added to the zirconia to produce ceria in tetragonal stabilized zirconia (Ce-TZP).

### Properties of zirconia

#### Physical properties

Zirconia is a stable restorative biomaterial. Dental zirconia is resistant to acid erosive attacks in the mouth although some erosive agents may have a negative effect on the surface roughness (Tanweer et al., 2022). It has extremely low thermal conductivity and the thermal expansion coefficient is 10 × 10 − 6/°C and does not depend on the yttria content (Ban, 2021).

#### Mechanical properties

Zirconia has the highest hardness among the various restorative materials used in dentistry (Ban, 2021). Its flexural strength and hardness are extremely large compared to other restorative materials. Conventional zirconia has higher bi-axial flexural strength compared to high-translucent monolithic zirconia (Kontonasaki, Giasimakopoulos & Rigos, 2020). Furthermore, the fracture toughness of 5Y-TZP is almost 50% less compared to that of 3Y-TZP with the cubic phase content because of more yttria content (Belli et al., 2021). In a recent study, (Liao et al., 2023) showed that the flexural strength value was 584 (158) MPa for 3Y-TZP and 373 (104) MPa for 5Y-TZP.

Dal Piva et al. (2018) studied the influence of the milling system and aging on zirconia surface roughness and phase transformation and they found that the surface roughness of zirconia-based crowns was not influenced by the milling system or low-temperature degradation. But regarding the phase transformation, autoclaving and pH-cycling aging presented a monoclinic phase increase when compared to the control group and thermocycled group. Similarly, a study by Flinn et al. (2012) on the accelerated aging of Y-TZP found that the hydrothermal aging of Y-TZP can cause a significant transformation from tetragonal to monoclinic crystal structure with a significant decrease in the flexural strength of thin bars. Hence, the aging of zirconia increases the monoclinic phase.

The fracture strength of a zirconia implant is influenced by its design, composition, and kind of abutment preparation (Bethke et al., 2020). The 1-piece zirconia implant fixture has twice the fracture strength compared to the 2-piece fixture (Kohal, Finke & Klaus, 2009). There is a strong correlation between the fracture toughness and fracture loads of ceramic crowns on zirconia implants during the occlusal contact (Rohr, Märtin & Fischer, 2018). Therefore, proper selection of zirconia material should be done for the crown whether aesthetics or strength is needed.

Zirconia is supposed to cause the opposing teeth to wear. But smooth and well-polished zirconia does not cause tooth wear. Abrasive wear on the occlusal part of zirconia restoration affects the opposing teeth or restoration (Mair, 1992). When the zirconia restoration is a hard and rough surface, the tooth abrasive wear becomes severe.

#### Optical properties

**Figure 3:**
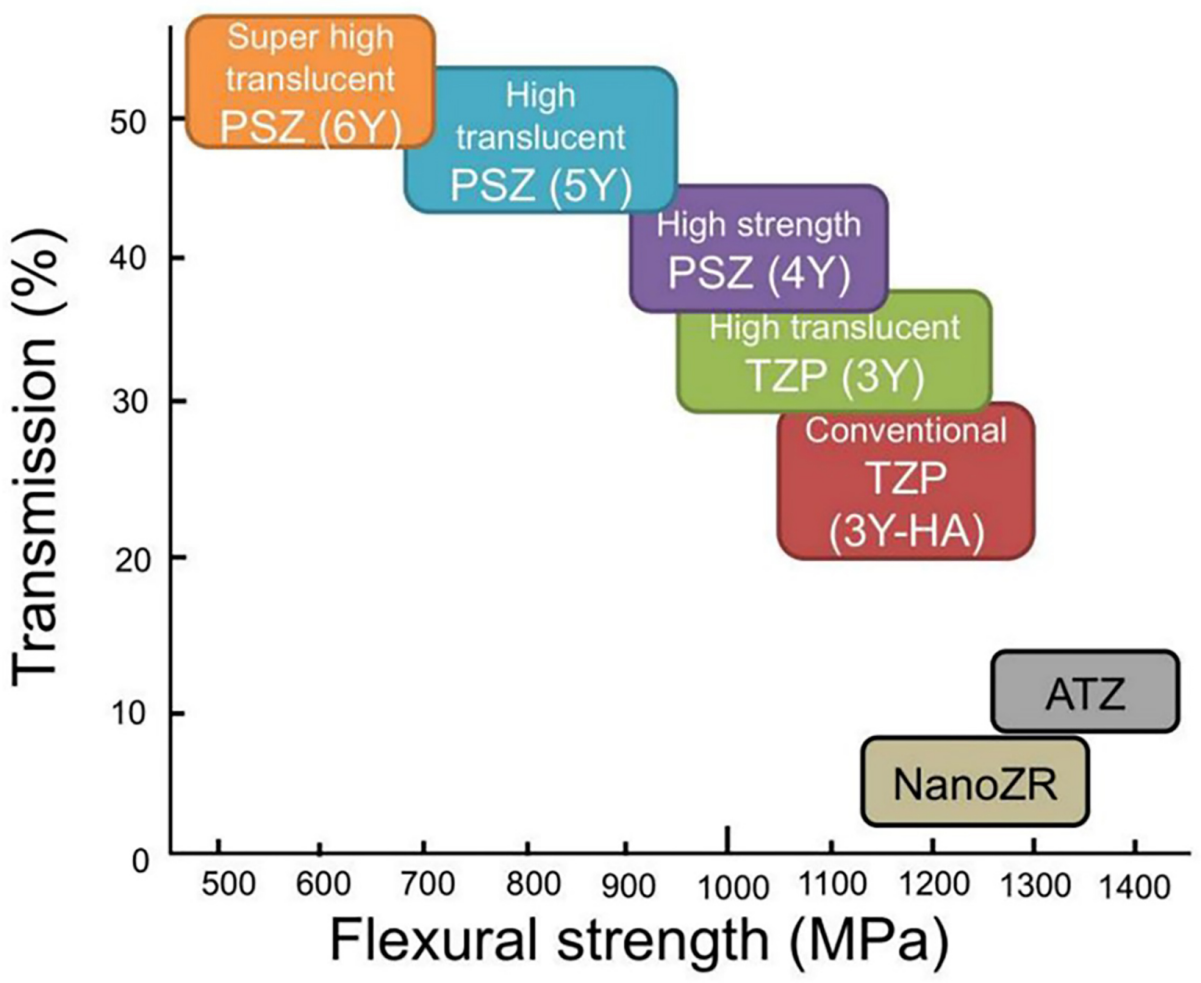
The translucency *vs* flexural strength of dental zirconia Ban (2021).

Zirconia is an esthetic biomaterial, but its translucency is slightly less compared to the glass-ceramics. To maintain the translucency of the zirconia and glass-ceramic prostheses, suitable luting cement should be used (Heboyan et al., 2023; Bilgrami et al., 2022b; Bilgrami et al., 2022a). The addition of yttria content in zirconia increases the cubic phases and this increases the translucency, however, the strength is reduced due to a few tetragonal phases (Fig. 3). 5Y-TZP is more translucent by 20 to 25% but has less flexural strength by 40 to 50% compared to 3Y (Ban, 2021). Hence, 3Y-TZP can be indicated for bridges, especially of long spans, and is not suitable for the anterior teeth (Ban, 2021; Liao et al., 2023). Conversely, 5Y-TZP and M5Y are indicated for veneers and anterior crowns but are not suitable for long-span bridges (Ban, 2021). Similarly, for the hybrid multilayer and polychromatic zirconia types, such as M3Y-5Y, their uses are similar to 5Y-TZP which has low strength (Jitwirachot et al., 2022). Similarly, both 4Y-TZP and M4Y can be used in all areas requiring sufficient strength and translucency.

Zirconia has greater radio-opacity compared to aluminum and titanium. This is due to its intrinsically high density and effective atoms which can obtain high-contrast radiographic images useful for diagnosis (Ban, 2021). Speed sintering can reduce the translucency of the zirconia. It was found that regular sintering had larger gain sizes and increased translucency than speed sintering (Kongkiatkamon & Peampring, 2022).

#### Biological properties

Various animal and human studies conclude that zirconia is a biocompatible biomaterial (Bapat et al., 2022; Josset et al., 1999; Christel et al., 1989; Uo et al., 2003; Abd El-Ghany & Sherief, 2016; Zarone et al., 2021). Christel et al. (1989) investigated the effect of yttria-stabilized zirconia and alumina *in vivo* (implanted into paraspinal muscles of rats) and found no cytotoxicity. Similarly, Josset et al. (1999) also found that the human osteoblasts presented good adhesion and cell spreading, and the cells maintained their proliferation capacity and differentiation ability into osteogenic pathways. Wu et al. (2015) studied the wettability of ZrO_2_ and found that its wettability was substantially enhanced by oxygen plasma treatment for maintaining a stable hydrophilicity surface. Water droplets can wet the hydrophilic zirconia surface (low contact angle) and this wetting condition that is suitable for oil-water separation is achieved by engineering the surface chemistry and surface roughness characteristics (Rasouli et al., 2021). Hydrophilic surface is an important factor that affects protein absorption and human gingival fibroblasts’ cellular attachment to implant abutments (Rutkunas et al., 2022; Barberi & Spriano, 2021; Kim et al., 2015) Generally, a lower contact angle promotes fibroblast attachment (Kim et al., 2015).

Furthermore, zirconia does not cause mutations in the cellular genome (Silva, Lameiras & Lobato, 2002; Warashina et al., 2003). Moreover, ZrO_2_ creates a less toxic reaction in tissue compared to titanium (Degidi et al., 2006). Zirconia also shows less bacterial adhesion and it is important in maintaining good periodontal health (Scarano et al., 2004). It was found that zirconia showed less adhesion of bacteria and less biofilm formation compared to titanium (Ban, 2021; Scarano et al., 2004; Rimondini et al., 2002). Scarano et al. (2004) found that bacterial adhesion was 12.1% on zirconia *vs* to 19.3% on titanium.

### Sintering of zirconia

CAD/CAM technology used computer-aided design and fabrication of ceramic prostheses and the process is more time efficient than conventional techniques (Padrós et al., 2020; Abduo & Lyons, 2013). Sintering is responsible for providing the strengths to the zirconia restoration. Various sintering methods have been developed and they affect the structure, properties, and esthetics of zirconia (Kilinc & Sanal, 2021; Juntavee & Attashu, 2018; Sanal & Kilinc, 2020). Different studies compared different (slow and fast) sintering protocols of zirconia (Amat et al., 2019; Kilinc & Sanal, 2021; Ordoñez Balladares et al., 2022; Ersoy et al., 2015; Liu et al., 2022b). Juntavee & Attashu (2018) studied the role of sintering duration and temperature on the mechanical properties of zirconia and found that a long sintering time with high sintering temperature results in increased flexural strength zirconia. Similarly, Kongkiatkamon & Peampring (2022) evaluated the surface microstructure, flexural strength, and translucency of 5Y-TZP zirconia using regular and speed sintering. They found that the regular protocol showed bigger gain sizes and more translucency than the speed protocol. The speed sintering had higher biaxial flexural strengths which can be due to changes in the material structure from the degradation of the metal salts (Sulaiman et al., 2017). Similarly, Liu et al. (2022a) also found that the Y-PSZ with conventional sintering had a bigger average grain size and fewer fine grains compared to the speed sintering of zirconia. Ahmed et al. (2020) found no dimensional change between normal and fast sintering of zirconia. Liu et al. (2022b) investigated the optical properties of 3Y-TZP and 5Y-TZP and noticed that speed sintering had less lightness without affecting the surface roughness.

### Surface treatment and adhesion of zirconia

Bonding between resin cement and zirconia is difficult to achieve because of their chemical inertness and lack of silica content (Scaminaci Russo et al., 2019). Hence, surface treatments of the zirconia restoration increase the adhesive, micro tensile bond strength, and longevity of the prosthetic restoration (Araújo et al., 2018; Melo et al., 2015; Heboyan et al., 2023). At present various surface treatments for zirconia and ceramics are available for better bonding to the tooth structure (Campos et al., 2016; Guarda et al., 2013; Sato et al., 2016). Airborne-particle abrasion and tribo-chemical silica coating are the pre-treatment methods. Adhesion can be increased after physicochemical conditioning of zirconia (Scaminaci Russo et al., 2019). One common treatment includes alumina particles followed by the application of primers or cement-based on10 MDP (methacryloyloxydecyl dihydrogen phosphate) (Melo et al., 2015; Aung et al., 2019; Shimizu et al., 2018; Silveira et al., 2022; Alammar & Blatz, 2022) However, the effect of the bond strength with the new generation of high-translucent zirconia materials is not clear and further studies are needed.

### Classification of zirconia

The previous classifications of zirconia were done according to the types of polycrystalline (zirconia, Alumina, PSZ, TZP, and yttria-stabilized dental zirconia; Generation 1–3) (Sato et al., 2016). Zirconia can be of various types as shown in Table 1. Commonly, zirconia can be uniform or hybrid in composition and monolayer or multilayer.

**Table 1:** Dental zirconia materials in the market.

**Zirconia**	**Yttria content** **(** **mol** **%)**	**Indications**
**A** **.** **Ammanngirrbach**		
*Super High Translucent* (*SHT*)		
1. Ceramill Zolid Fx White	5%	Anatomical crowns and bridges (<3 units extending to the molar region) Veneers, Inlays, Onlays
2. Ceramill Zolid Fx Multilayers	5%	
*High Translucent* (*HT*)		
1. Zolid gen x	4%	Anatomical crowns and 4- to multi-unit bridges
		Multi-unit screw-retained constructions on Ti bases
2. Zolid drs multilayer	4%	Crowns and bridges (<3 units up to molar region)
		Veneers, inlays, onlays
		Individual abutments
3. Zolid ht+ preshades	4%	Anatomical crowns and 4- to multi-unit bridges
		Multi-unit screw-retained constructions on Ti bases
4. Zolid ht+ white	4%	Anatomical crowns and 4- to multi-unit bridges
		Multi-unit screw-retained constructions on Ti bases
*Low Translucent* (*LT*)		
1. Ceramill Zi	3%	Custom abutments on titanium bases
		Crowns and 4-unit to multi-unit bridge frameworks
		Multi-unit, screw-retained restorations on titanium bases
**B** **.** **Vita YZ**		
1. YZ T	3%	Anatomical crowns and up to 14-unit bridges in the anterior and posterior tooth region
		Single-tooth and up to 14-unit bridges on screw-retained restorations in the anterior and posterior tooth region
		Primary telescopes
2. YZ HT	3%	Anatomical crowns and up to 14-unit bridges in the anterior and posterior tooth region
		Single-tooth and up to 14-unit bridges on screw-retained restorations in the anterior and posterior tooth region
		Primary telescopes
3. YZ ST	4%	Anatomical crowns and up to 14-unit bridges in the anterior and posterior tooth region
		Single-tooth and up to 14-unit bridges on screw-retained restorations in the anterior and posterior region
		Inlays, onlays, veneers, table top
4. YZ XT	5%	Anatomical single-tooth crowns and up to 3-unit bridges
		Inlays, onlays, veneers, table top
5. YZ ST Multicolors	4%	Anatomical crowns and up to 14-unit bridges in the anterior and posterior tooth region
		Single-tooth and up to 14-unit bridges on screw-retained restorations in the anterior and posterior tooth region
		Inlays, onlays, veneers, table top
6. YZ XT Multicolors	5%	Anatomical single-tooth crowns and up to 3-unit bridges
		Inlays, onlays, veneers, table top
**C** **.** **Cercon**		
1. Cercon base	3%	Anatomical crowns and up to 14-unit bridges in the anterior and posterior tooth region
2. Cercon ht	3%	Anatomical crowns and up to 14-unit bridges in the anterior and posterior tooth region
		Primary telescopes
3. Cercon xt	5%	Anatomical crowns and bridges (<3 units extending to the second premolar region)
4. Cercon ht ML	3%	Anatomical crowns and up to 14-unit bridges in the anterior and posterior tooth region
		Primary telescopes
5. Cercon xt ML	5%	Anatomical crowns and bridges (<3 units extending to the second premolar region)
**D** **.** **Lava 3M**		
1. Lava Plus	3%	Full-arch bridges
		Splinted crowns up to 4 units
		Primary telescopes
		Crowns (anterior and posterior)
2. Lava Esthetic	5%	3-unit bridges (<1 pontic between 2 crowns)
		Anterior and posterior crowns
3. Lava Chairside Zirconia	3%	Single crown
		3-unit bridges (<1 pontic between 2 crowns)
**E** **.** **GC Initial**		
1. Standard Translucency (ST)	3%	Anterior and posterior crown Hybrid abutment
2. High Translucency (HT)	3%	Implant framework Multi-unit bridge
3. Ultra High Translucency (UHT)	3%	Inlay, onlay, veneer Anatomical single-tooth crowns and up to 3-unit bridges
**F** **.** **Sagemax**		
1. NexxZr S: High Strength	3%	Single crown
		Frameworks up to multi-unit frameworks
2. NexxZr T: Translucent	3%	Single-unit restorations up to multi-unit bridges
3. NexxZr T Multi: Translucent	3% (cervical) & 5% (incisal)	Single-unit restorations up to multi-unit bridges
4. NexxZr+: Hight Translucent	4%	Single-unit restorations up to multi-unit bridges (white) or 3-unit bridges (preshaded)
5. NexxZr Multi: High Translucent	4% (cervical) & 5% (incisal)	
**G** **.** **Dental Direk**		
1. DD cubeX^2^^®^ –Super High Translucent (SHT)	5%	High esthetic monolithic crowns and bridges (<3 units, including molar restorations)
2. DD cube ONE^®^ –High Translucent Plus (HT+)	4%	High esthetic monolithic crowns and bridges (≥ 4 units) High esthetic veneering
3. DD Bio ZX^2^ –High Translucent (HT)	3%	Monolithic crowns and bridges (of any span range)
4. DD Bio Z –High Strength (HS)	3%	Monolithic crowns and bridges (of any span range)
		Implant superstructures
		Abutments
**H** **.** **Katana**		
1. LT	3%	Single-unit frameworks and long-span bridges
2. HT	3%	
3. HTML	3%	
4. STML	4%	Single-unit or <3-unit posterior bridges
5. UTML	5%	Anterior crowns and veneers, inlays/onlays, and posterior single crowns.
6. YML	3% (cervical) & 5% (incisal)	Veneers, Inlays, Onlays
		Single crown (Anterior and posterior), Longspan bridge,
		Framework
**I**. **Emax Zir CAD**		
1. MT Multi	4% (dentin) & 5% enamel)	Full contour crown, 3-unit bridge
2. MT	4%	Crown, 3-unit bridge, Implant-supported superstructures
3. LT	3%	Crown copings
		Multi-unit bridges with <2 pontics
4. MO	3%	Crown coping
		Multi-unit bridges with <2 pontics

**Figure 4:**
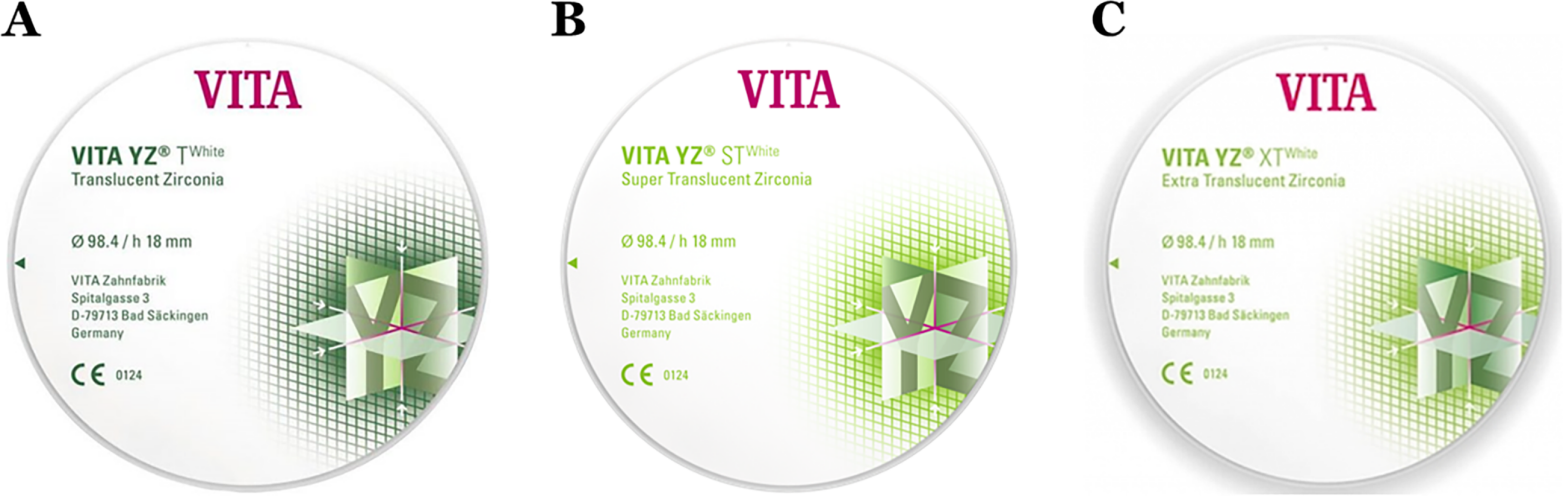
Examples of zirconia-based on the yttria content. A, 3Y-TZP, B, 4Y-TZP, and C, 5Y-TZP.

**Figure 5:**
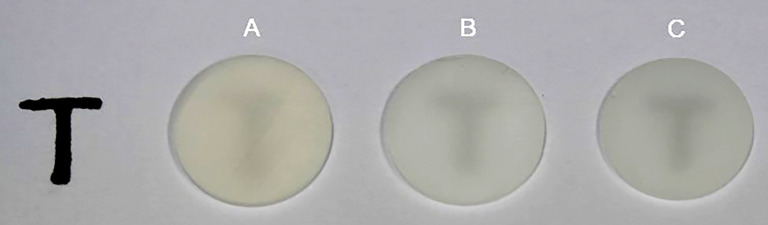
Translucency of zirconia-based on the yttria content. A, 3Y-TZP, B, 4Y-TZP, and C, 5Y-TZP.

Since there are various ceramic materials in the market and it is often confusion regarding choosing the material. Hence, the authors would like to categorize the zirconia materials based on their composition, and an indication of the commercially available zirconia materials (Table 2 and Figs. 4–5).

**Table 2:** Current classification of the zirconia-based on the yttria content and indications.

**Types**	**Indications**
**Type 1A**: **3Y-TZP(conventional)** (1) **Ceramill Zi** (2) **Vita YZ T** (3) **Cercon base** (4) **Kantana LT** (5) **Emax Zir CAD LT**	–Substructure –Custom abutment –Single-tooth and up to 14-unit bridges on screw-retained restorations in the anterior and posterior tooth region (primary telescopic)
**Type 1B**: **3Y-TZP with reduced alumina** (1) **Vita YZ HT** (2) **Cercon HT** (3) **Lava Plus** (4) **Lava chairside** (5) **GC Standard Translucency (ST)** (6) **GC High Translucency (HT)** (7) **GC Ultra High Translucency (UHT)** (8) **Nexx Zr S** (9) **Nexx Zr T** (10) **DD Bio Z High Strength (HS)** (11) **DD Bio ZX^2^ High Translucent (HT)** (12) **Katana HT** (13) **Katana HT ML** (14) **E Max Zr CAD MO**	–Substructure –Custom abutment –Single-tooth and up to 14-unit bridges on screw-retained restorations in the anterior and posterior tooth region (primary telescopic)
**Type 2: 4Y-TZP** (1) **Zolid gen x** (2) **Zolid drs multilayer** (3) **Zolid ht+ preshades** (4) **Zolid ht+ white** (5) **Vita YZ ST** (6) **Vita YZ ST Multicolor** (7) **NexxZr+: Hight Translucent** (8) **DD cube ONE^®^ –High Translucent Plus (HT+)** (9) **Katana STML** (10) **Emax Zircad MT**	–Single-tooth and up to 14-unit bridges on screw-retained restorations in the anterior and posterior region –Inlay, onlay, tabletop
**Type 3: 5Y-TZP** (1) **Ceramill Zolid Fx White** (2) **Ceramill Zolid Fx Multilayers** (3) **Vita YZ XT** (4) **YZ XT Multicolors** (5) **Cercon XT** (6) **Cercon XT ML** (7) **LAVA Esthetic** (8) **DD cubeX^2^^®^ –Super High Translucent (SHT)** (9) **Katana UTML**	− Anatomical crowns and bridges (<3 units extending to the second premolar region)
**Type 4: Combination of 3Y/ 4Y and 5Y-TZP** (1) **NexxZr T Multi: Translucent** (2) **NexxZr Multi: High Translucent** (3) **Katana YML** (4) **Emax Zir CAD MT Multi**	–Single unit –Multiple unit bridge

Finally, this review article provides updated information on the various dental types of zirconia used in dentistry. As this review does not use the PICO method, this review can be extended to do a more extensive review following the PICOS method.

## Conclusions

Zirconia can be of various types based on the yttria content, uniform or hybrid composition, monochromatic or polychromatic, and monolayer or multilayer. Increased yttria content in zirconia results in higher translucency but reduces the strength. Zirconia with lower yttria content (3Y-TZP, 3 mole % Y-TZP) has better mechanical properties and less translucency whereas 3Y-TZP (3 mole % Y-TZP) with increased yttria content (6Y-TZP, 6 mole % Y-TZP) has more translucency but presents lower mechanical properties. Speed sintering of zirconia has resulted in higher flexural strength and regular sintering of zirconia has shown bigger gain sizes and more translucency.

## Additional Information and Declarations

### Data Availability

The following information was supplied regarding data availability: This is a literature review.

## References

[ref-1] Abd El-Ghany OS, Sherief AH (2016). Zirconia based ceramics some clinical and biological aspects: review. Future Dental Journal.

[ref-2] Abdulmajeed A, Sulaiman T, Abdulmajeed A, Bencharit S, Närhi T (2020). Fracture load of different zirconia types: a mastication simulation study. Journal of Prosthodontics.

[ref-3] Abduo J, Lyons K (2013). Rationale for the use of CAD/CAM technology in implant prosthodontics. International Journal of Dentistry.

[ref-4] Ahmed N, Abbasi MS, Haider S, Ahmed N, Habib SR, Altamash S, Zafar MS, Alam MK (2021). Fit accuracy of removable partial denture frameworks fabricated with CAD/CAM, rapid prototyping, and conventional techniques: a systematic review. BioMed Research International.

[ref-5] Ahmed WM, Troczynski T, Stojkova BJ, McCullagh AP, Wyatt CC, Carvalho RM (2020). Dimensional changes of yttria-stabilized zirconia under different preparation designs and sintering protocols. Journal of Prosthodontics.

[ref-6] Al-Qahtani AS, Tulbah HI, Binhasan M, Abbasi MS, Ahmed N, Shabib S, Farooq I, Aldahian N, Nisar SS, Tanveer SA, Vohra F, Abduljabbar T (2021). Surface properties of polymer resins fabricated with subtractive and additive manufacturing techniques. Polymers.

[ref-7] Alammar A, Blatz MB (2022). The resin bond to high-translucent zirconia-a systematic review. Journal of Esthetic and Restorative Dentistry.

[ref-8] Alqutaibi AY, Ghulam O, Krsoum M, Binmahmoud S, Taher H, Elmalky W, Zafar MS (2022). Revolution of current dental zirconia: a comprehensive review. Molecules.

[ref-9] Amat NF, Muchtar A, Amril MS, Ghazali MJ, Yahaya N (2019). Effect of sintering temperature on the aging resistance and mechanical properties of monolithic zirconia. Journal of Materials Research and Technology.

[ref-10] Amornvit P, Rokaya D, Peampring C, Sanohkan S (2021). Confocal 3D optical intraoral scanners and comparison of image capturing accuracy. Computers, Materials & Continua.

[ref-11] Araújo AMM, Januário A, Moura DMD, Tribst JPM, Özcan M, Souza ROA (2018). Can the application of multi-mode adhesive be a substitute to silicatized/silanized Y-TZP ceramics?. Brazilian Dental Journal.

[ref-12] Arcila LVC, Ramos NC, Campos TMB, Dapieve KS, Valandro LF, de Melo RM, Bottino MA (2021). Mechanical behavior and microstructural characterization of different zirconia polycrystals in different thicknesses. Journal of Advanced Prosthodontics.

[ref-13] Aung S, Takagaki T, Lyann SK, Ikeda M, Inokoshi M, Sadr A, Nikaido T, Tagami J (2019). Effects of alumina-blasting pressure on the bonding to super/ultra-translucent zirconia. Dental Materials.

[ref-14] Ban S (2021). Classification and properties of dental zirconia as implant fixtures and superstructures. Materials.

[ref-15] Bapat RA, Yang HJ, Chaubal TV, Dharmadhikari S, Abdulla AM, Arora S, Rawal S, Kesharwani P (2022). Review on synthesis properties and multifarious therapeutic applications of nanostructured zirconia in dentistry. RSC Advances.

[ref-16] Barberi J, Spriano S (2021). Titanium and protein adsorption: an overview of mechanisms and effects of surface features. Materials.

[ref-17] Belli R, Hurle K, Schürrlen J, Petschelt A, Werbach K, Peterlik H, Rabe T, Mieller B, Lohbauer U (2021). A revised relationship between fracture toughness and Y2O3 content in modern dental zirconias. Journal of the European Ceramic Society.

[ref-18] Bethke A, Pieralli S, Kohal RJ, Burkhardt F, Stein-Lausnitz Mvon, Vach K, Spies BC (2020). Fracture resistance of zirconia oral implants in vitro: a systematic review and meta-analysis. Materials.

[ref-19] Bilgrami A, Alam MK, Qazi FuR, Maqsood A, Basha S, Ahmed N, Syed KA, Mustafa M, Shrivastava D, Nagarajappa AK, Srivastava KC (2022a). An in-vitro evaluation of microleakage in resin-based restorative materials at different time intervals. Polymers.

[ref-20] Bilgrami A, Maqsood A, Alam MK, Ahmed N, Mustafa M, Alqahtani AR, Alshehri A, Alqahtani AA, Alghannam S (2022b). Evaluation of shear bond strength between resin composites and conventional glass ionomer cement in class ii restorative technique—an in vitro study. Materials.

[ref-21] Bocanegra-Bernal MH, dela Torre SD (2002). Phase transitions in zirconium dioxide and related materials for high performance engineering ceramics. Journal of Materials Science.

[ref-22] Campos F, Almeida CS, Rippe MP, de Melo RM, Valandro LF, Bottino MA (2016). Resin bonding to a hybrid ceramic: effects of surface treatments and aging. Operative Dentistry.

[ref-23] Chevalier J (2006). What future for zirconia as a biomaterial?. Biomaterials.

[ref-24] Chevalier J, Cales B, Drouin JM (1999). Low-temperature aging of Y-TZP ceramics. Journal of the American Ceramic Society.

[ref-25] Cho YE, Lim YJ, Han JS, Yeo IL, Yoon HI (2020). Effect of yttria content on the translucency and masking ability of yttria-stabilized tetragonal zirconia polycrystal. Materials.

[ref-26] Christel P, Meunier A, Heller M, Torre JP, Peille CN (1989). Mechanical properties and short-term in-vivo evaluation of yttrium-oxide-partially-stabilized zirconia. Journal of Biomedical Materials Research.

[ref-27] Dal Piva AM, Tribst JP, Gondim LD, Ribeiro IL, Campos F, Arata A, Souza RO (2018). Y-TZP surface behavior under two different milling systems and three different accelerated aging protocols. Minerva Dental and Oral Science.

[ref-28] Degidi M, Artese L, Scarano A, Perrotti V, Gehrke P, Piattelli A (2006). Inflammatory infiltrate, and density, microvessel, nitric oxide synthase expression, vascular endothelial growth factor expression, and proliferative activity in peri-implant soft tissues around titanium and zirconium oxide healing caps. Journal of Periodontology.

[ref-29] Ersoy NM, Aydoğdu HM, Değirmenci B, Çökük N, Sevimay M (2015). The effects of sintering temperature and duration on the flexural strength and grain size of zirconia. Acta Biomaterialia Odontologica Scandinavica.

[ref-30] Flinn BD, de Groot DA, Mancl LA, Raigrodski AJ (2012). Accelerated aging characteristics of three yttria-stabilized tetragonal zirconia polycrystalline dental materials. Journal of Prosthetic Dentistry.

[ref-31] Grech J, Antunes E (2019). Zirconia in dental prosthetics: a literature review. Journal of Materials Research and Technology.

[ref-32] Guarda GB, Correr AB, Gonçalves LS, Costa AR, Borges GA, Sinhoreti MA, Correr-Sobrinho L (2013). Effects of surface treatments, thermocycling, and cyclic loading on the bond strength of a resin cement bonded to a lithium disilicate glass ceramic. Operative Dentistry.

[ref-33] Güth JF, Stawarczyk B, Edelhoff D, Liebermann A (2019). Zirconia and its novel compositions: what do clinicians need to know?. Quintessence International.

[ref-34] Harada A, Shishido S, Barkarmo S, Inagaki R, Kanno T, Örtengren U, Egusa H, Nakamura K (2020). Mechanical and microstructural properties of ultra-translucent dental zirconia ceramic stabilized with 5 mol% yttria. Journal of the Mechanical Behavior of Biomedical Materials.

[ref-35] Heboyan A, Vardanyan A, Karobari MI, Marya A, Avagyan T, Tebyaniyan H, Mustafa M, Rokaya D, Avetisyan A (2023). Dental luting cements: an updated comprehensive review. Molecules.

[ref-36] Humagain M, Rokaya D (2019). Integrating digital technologies in dentistry to enhance the clinical success. Kathmandu University Medical Journal.

[ref-37] Jitwirachot K, Rungsiyakull P, Holloway JA, Jia-Mahasap W (2022). Wear behavior of different generations of zirconia: present literature. International Journal of Dentistry.

[ref-38] Josset Y, Oum’Hamed Z, Zarrinpour A, Lorenzato M, Adnet JJ, Laurent-Maquin D (1999). In vitro reactions of human osteoblasts in culture with zirconia and alumina ceramics. Journal of Biomedical Materials Research.

[ref-39] Juntavee N, Attashu S (2018). Effect of different sintering process on flexural strength of translucency monolithic zirconia. Journal of Clinical and Experimental Dentistry.

[ref-40] Kilinc H, Sanal FA (2021). Effect of sintering and aging processes on the mechanical and optical properties of translucent zirconia. Journal of Prosthetic Dentistry.

[ref-41] Kim YS, Shin SY, Moon SK, Yang SM (2015). Surface properties correlated with the human gingival fibroblasts attachment on various materials for implant abutments: a multiple regression analysis. Acta Biomaterialia Odontologica Scandinavica.

[ref-42] Kohal RJ, Finke HC, Klaus G (2009). Stability of prototype two-piece zirconia and titanium implants after artificial aging: an in vitro pilot study. Clinical Implant Dentistry and Related Research.

[ref-43] Kongkiatkamon S, Booranasophone K, Tongtaksin A, Kiatthanakorn V, Rokaya D (2021). Comparison of fracture load of the four translucent zirconia crowns. Molecules.

[ref-44] Kongkiatkamon S, Peampring C (2022). Effect of speed sintering on low temperature degradation and biaxial flexural strength of 5Y-TZP zirconia. Molecules.

[ref-45] Kontonasaki E, Giasimakopoulos P, Rigos AE (2020). Strength and aging resistance of monolithic zirconia: an update to current knowledge. Japanese Dental Science Review.

[ref-46] Kontonasaki E, Rigos AE, Ilia C, Istantsos T (2019). Monolithic zirconia: an update to current knowledge, optical properties, wear, and clinical performance. Dentistry Journal.

[ref-47] Leib EW, Vainio U, Pasquarelli RM, Kus J, Czaschke C, Walter N, Janssen R, Müller M, Schreyer A, Weller H, Vossmeyer T (2015). Synthesis and thermal stability of zirconia and yttria-stabilized zirconia microspheres. Journal of Colloid and Interface Science.

[ref-48] Liao Y, Gruber M, Lukic H, McLees J, Chen S, Boghosian A, Megremis S (2023). Survey of the mechanical and physical behaviors of yttria-stabilized zirconia from multiple dental laboratories. JADA Foundational Science.

[ref-49] Liu H, Inokoshi M, Nozaki K, Shimizubata M, Nakai H, Cho Too TD, Minakuchi S (2022a). Influence of high-speed sintering protocols on translucency, mechanical properties, microstructure, crystallography, and low-temperature degradation of highly translucent zirconia. Dental Materials.

[ref-50] Liu Y-C, Lin T-H, Lin Y-Y, Hu S-W, Liu J-F, Yang C-C, Yan M (2022b). Optical properties evaluation of rapid sintered translucent zirconia with two dental colorimeters. Journal of Dental Sciences.

[ref-51] Mair LH (1992). Wear in dentistry—current terminology. Journal of Dentistry.

[ref-52] Melo RM, Souza RO, Dursun E, Monteiro EB, Valandro LF, Bottino MA (2015). Surface treatments of zirconia to enhance bonding durability. Operative Dentistry.

[ref-53] Miyazaki T, Nakamura T, Matsumura H, Ban S, Kobayashi T (2013). Current status of zirconia restoration. Journal of Prosthodontic Research.

[ref-54] Nistor L, Grădinaru M, Rîcă R, Mărăešcu P, Stan M, Manolea H, Ionescu A, Moraru I (2019). Zirconia use in dentistry - manufacturing and properties. Current Health Sciences Journal.

[ref-55] Ordoñez Balladares A, Abad-Coronel C, Ramos JC, Martín Biedma BJ (2022). Fracture resistance of sintered monolithic zirconia dioxide in different thermal units. Materials.

[ref-56] Padrós R, Giner L, Herrero-Climent M, Falcao-Costa C, Ríos-Santos JV, Gil FJ (2020). Influence of the CAD-CAM systems on the marginal accuracy and mechanical properties of dental restorations. International Journal of Environmental Research and Public Health.

[ref-57] Piconi C, Maccauro G (1999). Zirconia as a ceramic biomaterial. Biomaterials.

[ref-58] Rasouli S, Rezaei N, Hamedi H, Zendehboudi S, Duan X (2021). Superhydrophobic and superoleophilic membranes for oil-water separation application: a comprehensive review. Materials & Design.

[ref-59] Rekow ED, Silva NRFA, Coelho PG, Zhang Y, Guess P, Thompson VP (2011). Performance of dental ceramics: challenges for improvements. Journal of Dental Research.

[ref-60] Rimondini L, Cerroni L, Carrassi A, Torricelli P (2002). Bacterial colonization of zirconia ceramic surfaces: an in vitro and in vivo study. International Journal of Oral and Maxillofacial Implants..

[ref-61] Rohr N, Märtin S, Fischer J (2018). Correlations between fracture load of zirconia implant supported single crowns and mechanical properties of restorative material and cement. Dental Materials Journal.

[ref-62] Rutkunas V, Borusevicius R, Balciunas E, Jasinskyte U, Alksne M, Simoliunas E, Zlatev S, Ivanova V, Bukelskiene V, Mijiritsky E (2022). The effect of UV treatment on surface contact angle, fibroblast cytotoxicity, and proliferation with two types of zirconia-based ceramics. International Journal of Environmental Research and Public Health.

[ref-63] Sanal FA, Kilinc H (2020). Do different sintering conditions influence bond strength between the resin cements and a currently used esthetic zirconia?. Journal of Adhesion Science and Technology.

[ref-64] Saridag S, Tak O, Alniacik G (2013). Basic properties and types of zirconia: an overview. World Journal of Stomatology.

[ref-65] Sato TP, Anami LC, Melo RM, Valandro LF, Bottino MA (2016). Effects of surface treatments on the bond strength between resin cement and a new zirconia-reinforced lithium silicate ceramic. Operative Dentistry.

[ref-66] Scaminaci Russo D, Cinelli F, Sarti C, Giachetti L (2019). Adhesion to zirconia: a systematic review of current conditioning methods and bonding materials. Dentistry Journal.

[ref-67] Scarano A, Piattelli M, Caputi S, Favero GA, Piattelli A (2004). Bacterial adhesion on commercially pure titanium and zirconium oxide disks: an in vivo human study. Journal of Periodontology.

[ref-68] Shimizu H, Inokoshi M, Takagaki T, Uo M, Minakuchi S (2018). Bonding efficacy of 4-META/MMA-TBB resin to surface-treated highly translucent dental zirconia. Journal of Adhesive Dentistry.

[ref-69] Silva VV, Lameiras FS, Lobato ZI (2002). Biological reactivity of zirconia-hydroxyapatite composites. Journal of Biomedical Materials Research.

[ref-70] Silveira MPM, Ramos NDC, Lopes GdRS, Tribst JPM, Bottino MA (2022). Bond strength between different zirconia-based ceramics and resin cement before and after aging. Coatings.

[ref-71] Sorrentino R, Navarra CO, Di Lenarda R, Breschi L, Zarone F, Cadenaro M, Spagnuolo G (2019). Effects of finish line design and fatigue cyclic loading on phase transformation of zirconia dental ceramics: a qualitative micro-raman spectroscopic analysis. Materials.

[ref-72] Sulaiman TA, Abdulmajeed AA, Shahramian K, Lassila L (2017). Effect of different treatments on the flexural strength of fully versus partially stabilized monolithic zirconia. Journal of Prosthetic Dentistry.

[ref-73] Tanweer N, Qazi Das F-U-R, Das G, Bilgrami A, Basha S, Ahmed N, Bahammam HA, Bahammam SA, Basheer SN, Assiry AA, Karobari MI, Khan AS, Heboyan A (2022). Effect of erosive agents on surface characteristics of nano-fluorapatite ceramic: an in-vitro study. Molecules.

[ref-74] Uo M, Sjögren G, Sundh A, Watari F, Bergman M, Lerner U (2003). Cytotoxicity and bonding property of dental ceramics. Dental Materials.

[ref-75] Warashina H, Sakano S, Kitamura S, Yamauchi KI, Yamaguchi J, Ishiguro N, Hasegawa Y (2003). Biological reaction to alumina, zirconia, titanium and polyethylene particles implanted onto murine calvaria. Biomaterials.

[ref-76] Wu CC, Wei CK, Ho CC, Ding SJ (2015). Enhanced hydrophilicity and biocompatibility of dental zirconia ceramics by oxygen plasma treatment. Materials.

[ref-77] Zarone F, Ruggiero G, Leone R, Breschi L, Leuci S, Sorrentino R (2021). Zirconia-reinforced lithium silicate (ZLS) mechanical and biological properties: a literature review. Journal of Dentistry.

[ref-78] Zhang Y (2014). Making yttria-stabilized tetragonal zirconia translucent. Dental Materials.

